# Romanian Inventory of Depression and Anxiety Symptoms (IDAS-II)

**DOI:** 10.3389/fpsyg.2023.1159380

**Published:** 2023-07-06

**Authors:** Ligiana Mihaela Petre, Delia Alexandra Gheorghe, David Watson, Laurentiu Mitrofan

**Affiliations:** ^1^Department of Applied Psychology and Psychotherapy, Faculty of Psychology and Educational Sciences, University of Bucharest, Bucharest, Romania; ^2^Department of Experimental and Theoretical Neuroscience, Transylvanian Institute of Neuroscience, Cluj-Napoca, Romania; ^3^Department of Psychology, University of Notre Dame, Notre Dame, IN, United States

**Keywords:** assessment, HiTOP, depressive disorders, anxiety disorders, bipolar disorder, posttraumatic stress disorder, obsessive-compulsive disorder, IDAS-II

## Abstract

**Background:**

The Inventory of Depression and Anxiety Symptoms (IDAS-II) is a self-report measure comprising 99 items divided into 18 non-overlapping scales that allows for a dimensional assessment of depression, anxiety, and bipolar symptoms. The IDAS-II is currently available in English, Turkish, Spanish, German, and Swedish. This study’s major goal was to adapt and validate the IDAS-II to the Romanian population.

**Method:**

Participants from a community sample (*N* = 1,072) completed the IDAS-II (Romanian version) and additional measures assessing depression and anxiety disorders.

**Results:**

Item-level factor analyses validated the unidimensionality of the scales, and internal consistency results indicated that most symptom scales had satisfactory alpha coefficient values. Based on previous structural analyses, a confirmatory factor analysis (CFA) on the IDAS-II scales confirmed a three-component model of “Distress,” “Obsessions/Fear,” and “Positive Mood.” Convergent and discriminant validity were established by correlational analyses with other symptom measures.

**Limitations:**

This study was conducted using a sample from the general population and several of the employed measures have limitations. Specifically, the current study was unable to employ Romanian versions of the gold-standard instruments that assess well-being, obsessive–compulsive disorder, and claustrophobia.

**Conclusion:**

The IDAS-II (Romanian version) is the first clinical measure to assess internalizing dimensions of the Hierarchical Taxonomy of Psychopathology (HiTOP) model that is available for the Romanian population.

## Introduction

The alarming prevalence, functional impairments, global burden, and substantial costs associated with depressive and anxiety disorders (Donohue and Pincus, 2007; Ferrari et al., 2013; Baxter et al., 2014; Stasik-O’Brien et al., 2019) should spur research efforts aimed at improving their identification, prevention, and treatment. One critical question that still needs to be addressed to advance the assessment of depressive and anxiety disorders pertains to the problematic reliability and validity of *the Diagnostic and Statistical Manual of Mental Disorders* (DSM; American Psychiatric Association, 1994) diagnoses (Chmielewski et al., 2015). Furthermore, the limits of traditional categorical nosologies, such as the DSM (American Psychiatric Association, 2022) and the *International Statistical Classification of Diseases and Related Health Problems* (11th ed.; *ICD-11*; World Health Organization, 2018) put significant constraints on the utility of categorical diagnoses for assessment and applied research (Kotov et al., 2017, 2021; Conway et al., 2022). Problems with categorical nosologies include the unreliability and low stability of diagnoses over time and between clinicians, rampant comorbidities, and excessive within-diagnosis heterogeneity (Conway et al., 2022). As a result, patients may often receive diagnoses of Other Specified/Unspecified Disorders that limit the development of a sufficiently detailed picture of the clinical condition (Verheul and Widiger, 2004; Dalle Grave and Calugi, 2007; Machado et al., 2007; Kotov et al., 2021). Toward this end, a taxonomy that addresses the limitations of categorical diagnoses is vital for assessment, treatment, and applied research (Clark et al., 2017).

To address this need, the Hierarchical Taxonomy of Psychopathology (HiTOP) consortium formulated a new nosological system based on structural evidence of continuity between psychopathology and normality (Krueger et al., 2018; Haslam et al., 2020). The HiTOP model reflects a paradigmatic break from traditional classification schemes and conceptualizes psychopathology in terms of continuously distributed dimensions, an approach that is supported by a large body of evidence (Haslam et al., 2020; Conway et al., 2022). Three fundamental findings guided HiTOP (Conway et al., 2022). First, dimensional description increases reliability (Shea et al., 2002; Markon et al., 2011; Narrow et al., 2013) and eliminates the requirement for Other Specified/Unspecified diagnoses, as every person has a standing on each dimension (Conway et al., 2022).

Second, as noted, many diagnoses are heterogeneous and encompass diverse characteristics. This problem is exacerbated by the fact that current nosological systems make ample use of polythetic diagnoses, such that a patient only needs to meet a specified number of criteria to have a disorder (e.g., 5 of 9 criteria to be diagnosed with major depression; see Watson et al., 2022). The HiTOP model addresses heterogeneity by decomposing broader syndromes into homogeneous dimensions (e.g., insomnia, suicidality) at lower levels of the hierarchical structure (Conway et al., 2019, 2022; Kotov et al., 2021; Watson et al., 2022).

Third, HiTOP classifies psychopathology hierarchically, from narrow to broad dimensions. Symptom components and maladaptive features are combined into dimensional syndromes, subfactors, and spectra. Superspectra include the general psychopathology factor (p factor) and predicted emotional dysfunction, psychosis, and externalizing symptoms (Kotov et al., 2017, 2020, 2021; Krueger et al., 2018, 2021; Watson et al., 2022). This hierarchy handles the problem of comorbidity because higher-order dimensions are derived from comorbidity patterns. Therefore, researchers and practitioners can use higher-order dimensions to emphasize commonalities or lower-order dimensions to model specific features (Krueger et al., 2018; Kotov et al., 2021; Conway et al., 2022).

The validated measures for implementing the HiTOP system could make diagnostic classification more effective in both research and clinical practice (Watson et al., 2022). The Inventory of Depression and Anxiety Symptoms-II (IDAS-II) measures a broad range of internalizing spectrum symptoms within the HiTOP paradigm (Kotov et al., 2017).

### The inventory of depression and anxiety symptoms

The inventory of depression and anxiety symptoms (IDAS-II), a factor analysis-based self-report measure of depression, anxiety, and bipolar symptoms, is congruent with the HiTOP structure of internalizing disorders. The IDAS-II has shown considerable evidence of reliability and validity across various samples, including children, adolescents, and older adults (Stasik-O’Brien et al., 2019; Weitzner et al., 2020; Cervin et al., 2023). The Romanian version of the IDAS-II accompanies other studies aimed at adapting HiTOP model to the Romanian population (Lozovanu et al., 2019; Constantin et al., 2021).

Watson et al. (2007) developed the first version of the IDAS (IDAS-I), which included 11 scales aimed at assessing specific depressive and anxiety symptoms consistent with the HiTOP. Subsequently, Watson et al. (2012) brought forth an Expanded Version of the IDAS (IDAS-II) that included 18 specific symptom scales, as well as a General Depression scale, which uses items from other IDAS-II scales and is intended to provide an overall depression symptom score. The expanded IDAS also assesses symptoms of bipolar disorder, obsessive–compulsive disorder (OCD), posttraumatic stress disorder (PTSD), social anxiety disorder (SAD), and claustrophobia. Both versions provide a dimensional assessment of each depression and anxiety symptom. This information helps clinicians and researchers determine how symptoms affect patients (Fried and Nesse, 2014, 2015).

Psychometric research has shown that the IDAS-II scales display satisfactory internal consistency and test–retest correlations (Watson and O’Hara, 2017). The General Depression and Dysphoria scales have a substantial effect size when comparing individuals with clinically significant psychopathology to normative population scores. Stasik-O’Brien et al. (2019) showed good to exceptional sensitivity, specificity, and area under the curve (AUC) values in receiver operating characteristic analysis for several IDAS scales in discriminating DSM-IV clinical diagnoses.

Based on exploratory and confirmatory factor analyses of the 18 non-overlapping scales of the IDAS-II, three factors were identified as defining its underlying structure (Watson et al., 2012; Irak and Albayrak, 2020): (a) a Distress factor, made up of scales that assess symptoms of major depressive disorder, generalized anxiety disorder, and post-traumatic stress disorder, corresponding to HiTOP’s Distress factor; (b) an Obsessions/Fear factor, made up of scales that assess symptoms of SAD, specific phobia, and OCD, corresponding to HiTOP’s Fear factor; (c) and a Positive Mood factor, composed of scales that assess symptoms of bipolar disorder, roughly corresponding to HiTOP’s Mania factor. Psychometric evidence suggests that the IDAS-II can measure HiTOP model-based internalizing spectrum symptoms (Kotov et al., 2017). Currently, the IDAS-II is available in the original English version (Watson et al., 2012) and has recently been adapted to Turkish (Irak and Albayrak, 2020), Spanish (de la Rosa-Cáceres et al., 2020), German (Wester et al., 2022), and Swedish (Cervin et al., 2023) populations.

The IDAS-II can be a useful tool to gather evidence on the structure of internalizing dimensions within the HiTOP model and to explore comorbidity between emotional disorders. The IDAS-II assesses a range of symptoms related to depression and anxiety, which are key components of the internalizing spectrum within the HiTOP approach. Moreover, it assesses them using same instructions, response format, and time frame, thereby eliminating problems related to method effects. Consequently, researchers can examine the patterns and relationships among the various symptoms and dimensions of internalizing psychopathology. The IDAS-II provides scores on specific scales that capture different facets of depression and anxiety, allowing for a more nuanced understanding of the internalizing spectrum. Additionally, the IDAS-II can highlight the comorbidity between emotional disorders as it assesses symptoms of both depression and anxiety simultaneously. By analyzing the co-occurrence and interrelationships of symptoms across these scales, researchers can gain insights into the comorbidity patterns within the internalizing domain. Furthermore, by using the IDAS-II within the context of HiTOP, researchers can investigate how the symptoms and dimensions are aligned with the hierarchical structure proposed by HiTOP. This can contribute to validating and refining the HiTOP model by providing empirical evidence for the organization of internalizing dimensions and their interrelations. In addition, the available data indicate that the IDAS-II has clear advantages in clinical contexts. It is useful for designing and assessing transdiagnostic interventions because it assesses the symptoms of multiple disorders that make up the internalizing dimensions of the HiTOP model. Assessment using IDAS-II facilitates clinical judgment and decision-making due to the norms and cutoffs provided to discriminate between individuals with and without a diagnosis (Stasik-O’Brien et al., 2019; Sanchez-Garcia et al., 2021; Wester et al., 2022), as well as between various levels of impairment (De la Rosa-Cáceres et al., 2023).

### Purpose of the present study

The present study adapted the IDAS-II to the Romanian-speaking population. Therefore, this study presents the psychometric properties of the Romanian version of the IDAS-II. With regards to reliability, high internal consistency and test**–**retest stability were expected, in line with previous studies (Watson et al., 2012; Watson and O’Hara, 2017; de la Rosa-Cáceres et al., 2020; Irak and Albayrak, 2020; Wester et al., 2022). As for convergent validity, strong correlations were predicted between scores on the IDAS-II scales and those on other measures of depression, anxiety, bipolar symptoms, and PTSD (Watson et al., 2007, 2012; Watson and O’Hara, 2017; de la Rosa-Cáceres et al., 2020; Irak and Albayrak, 2020; Wester et al., 2022). Finally, it was expected that the internal structure of the Romanian version of the IDAS-II would reflect three factors, similar to the English, Turkish, Spanish, and German versions (Watson et al., 2012; de la Rosa-Cáceres et al., 2020; Irak and Albayrak, 2020; Wester et al., 2022).

## Methods

### Sample

Participants were recruited across Romania via social media and asked to complete an online survey consisting of the four questionnaires listed below. There was no time limit and response variability was checked at random. Data collection was carried out from 8 December 2021 to 26 January 2022. Participants were briefed at the outset of the survey about the purpose of the study (to adapt a measure of anxiety and depression to the Romanian population) and the duration of the testing (on average, 30 min). Participants then provided informed consent online. Participants who opted to retake the testing provided their email address. Seven to 10 days following the initial submission, they received an invitation via Google Forms to fill out the survey once more. As a result, 154 participants comprised the convenience sample used to assess test–retest reliability.

Participants provided comprehensive demographic information, including their birth month and year, gender, ethnicity, place of residence, education level, occupation, household income range, marital status, partnership status, number of children, and health status. The final sample consisted of 1,064 participants, of whom 89.9% identified as female (*N* = 938) and 10.1% as male (*N* = 106). Age ranged between 19 and 65 (*M* = 25.1, *SD* = 8.73). Among participants, 3.76% had lower secondary education, 2.63% had post-secondary non-tertiary education, 57.14% had upper secondary education, 22.37% attained a Bachelor’s degree, 13.06% attained a Master’s degree, 0.75% attained a PhD, and 0.28% attained a post-doctorate’s degree. With respect to ethnicity, 96.4% participants were of Romanian ethnicity, 1.5% of Romanian–Hungarian mixed ethnicity, 0.66% of Romanian–Roma mixed ethnicity, 0.28% of Romanian–Russian–Lippovan-mixed ethnicity, 0.28% of Romanian–Macedonian mixed ethnicity, and 0.57% of another ethnicity.

Participants’ places of residence were divided into two groups: metropolitan areas (76.22%) and rural areas (23.78%). Regarding partnership status, 60.24% of the participants had a partner, while 39.76% were single. In terms of marital status, 13.82% were married, 81.67% unmarried, 0.75% separated, 3.76% divorced, and.09% were widowed. 85.24% of participants had no children and 14.75 had one, two or three children. As for occupation, 70.02% were students, 25.19% employed and 4.8% unemployed. 38.75% had an income higher than the minimum wage, 44.42% an income lower than the minimum wage, 6.8% the minimum wage, and 10.02% had no income. 84.96% of the sample had no acute or chronic illness diagnosis, while 15.04% did report an illness.

### Instruments

#### Inventory of depression and anxiety symptoms

Three authors translated the IDAS-II items and instructions and then compared their translations. Translators had the same translation for 72 of 99 items. For the remaining 27 items, translators discussed and agreed on a consensus version. This final Romanian version was backtranslated to English by an English native speaker who specialized in clinical psychology. Items in the back-translated version were found to be appropriately analogous to those of the original version.

The IDAS-II is composed of 99 items on a 5-point Likert scale, ranging from 1 (*not at all*) to 5 (*extremely*). Respondents rated the severity of their symptoms during the previous 2 weeks. The IDAS-II comprises a broad scale, General Depression, which contains items from several other scales, as well as 18 nonoverlapping scales (Dysphoria, Lassitude, Insomnia, Suicidality, Appetite Loss, Appetite Gain, Well-Being, Ill Temper, Mania, Euphoria, Panic, Social Anxiety, Claustrophobia, Traumatic Intrusions, Traumatic Avoidance, Checking, Ordering, and Cleaning). It thus provides extensive coverage of homogeneous symptom dimensions that underlie major depression, bipolar disorders, PTSD, panic disorder, agoraphobia, SAD, specific phobia, and OCD (Watson et al., 2012). The General Depression scale combines items from specific IDAS-II scales to give an overall depression symptom score, similar to the Beck Depression Inventory-II (BDI-II). As mentioned, the 18 scales have been found to reflect three main factors: distress, obsessions/fear, and positive mood (Watson et al., 2012).

#### Beck depression inventory-II

The Romanian version of the BDI-II (Beck et al., 1988; David et al., 2012) was used to measure cognitive and emotional symptoms of depression experienced during the previous week. In this study, this instrument was found to have strong internal consistency (Cronbach’s alpha = 0.97).

#### Beck anxiety inventory

Anxiety symptoms were assessed with the Romanian translation of the Beck Anxiety Inventory (BAI), developed by Beck et al. (1988). The BAI evaluates physical, emotional, and cognitive aspects of anxiety, as well as the fear of losing control (Beck et al., 1988). The BAI demonstrated strong internal consistency in this study, with a Cronbach’s alpha value of 0.95.

#### Millon clinical multiaxial inventory-III

A 175-item, true-false self-report measure, the Romanian version of the Millon Clinical Multiaxial Inventory-III (MCMI-III) was used to measure clinical personality and mental syndromes (Millon, 1997; Millon et al., 2010). The MCMI-III contains 24 clinical scales, arranged into four distinct categories: clinical personality patterns, severe personality pathology, clinical syndromes, and severe clinical syndromes. The Romanian version of the MCMI-III Clinical Syndromes scales (Anxiety, Somatoform, Mania, Dysthymia, Alcohol Dependence, Post-Traumatic Stress scales) exhibited good levels of internal consistency, ranging from a coefficient α of 0.71 (Bipolar scale) to 0.89 (Post-Traumatic Stress scale). As shown by Millon et al. (2010), retest intervals of 5 days to 4 months have provided a median reliability value across the Clinical Syndromes scales of *r* = 0.91, ranging from 0.84 (Anxiety scale) to 0.96 (Somatoform scale).

### Data analysis

Descriptive statistics were calculated. Internal consistency and item discrimination were measured using Cronbach’s alpha coefficient, average inter-item correlations (AICs), and Pearson correlations between scales. The unidimensionality of the scales was assessed using Horn’s parallel analysis and the point of inflection in scree plots (Dinno, 2009). Although Likert scales are inherently ordinal level data, parallel analyses are considered robust to non-normality (Braeken and Van Assen, 2017). Short-term test–retest stability was calculated using Pearson correlations between participants’ results over seven to 10 days. Pearson correlation coefficients were used to test convergent validity between IDAS-II scale scores and the other measures (i.e., BDI-II, BAI, MCMI-III).

**Figure 1:**
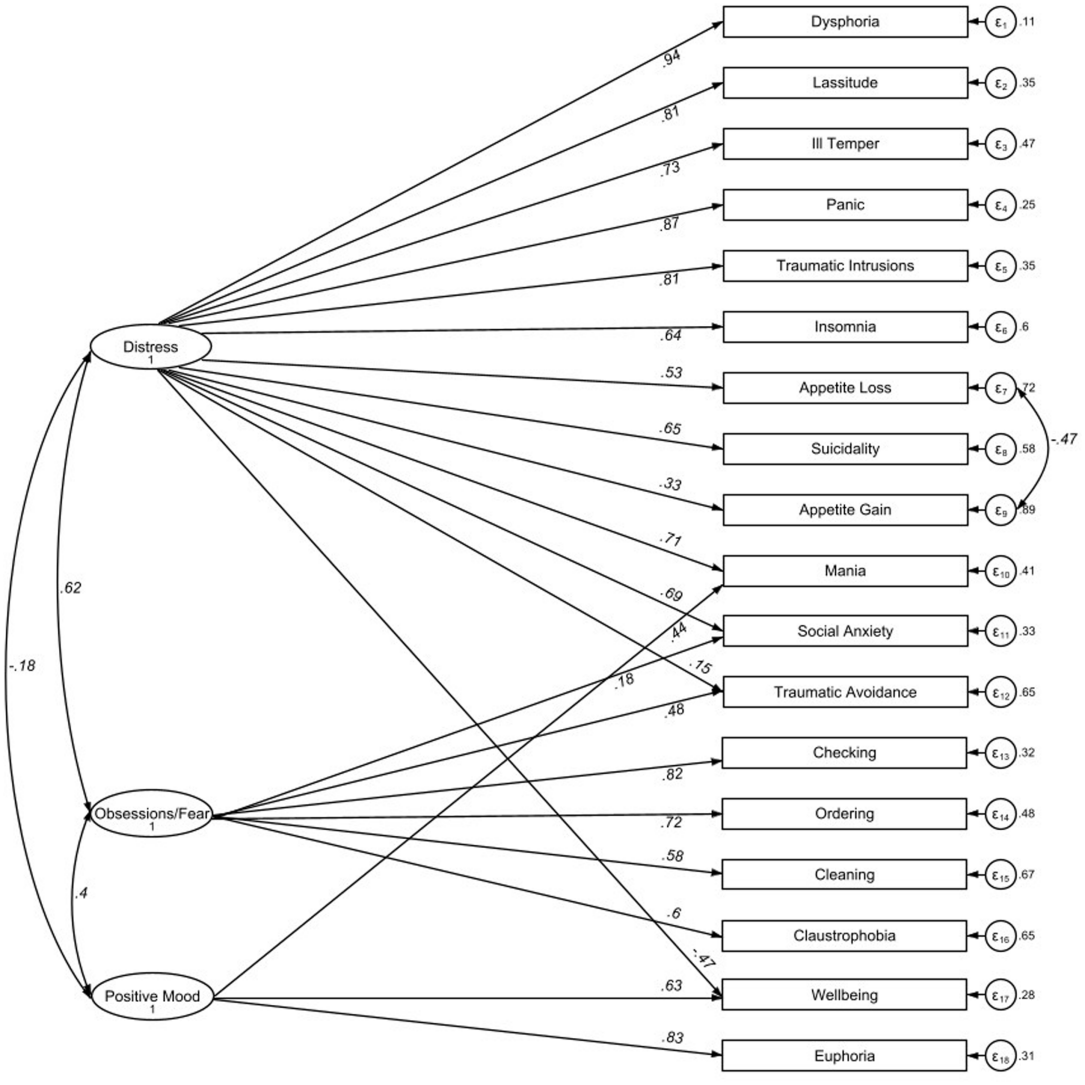
Path diagrams and factor loading. Values in italics show factor loadings as std. β coefficients; *N* = 1,064.

The internal structure of the instrument was examined using confirmatory factor analysis (CFA) by testing the three-factor structure proposed by Watson et al. (2012): distress, obsessions/fear, and positive mood (see Table 4 in Watson et al., 2012). Similar to the original paper, we allowed cross-loadings between factors (Watson et al., 2012). Specifically, Traumatic Avoidance was included in the Distress and Obsessions/Fear Factors, Mania was included in the Distress and Positive Mood Factors, Social Anxiety was included in the Distress and Obsessions/Fear factors; and Well-Being was included in the Distress and Positive Mood factors (Figure 1). Note that Mania and Well-Being may exhibit shared pathological components with Positive Mood (Well-Being) and Distress (Dysphoria), respectively, through demonstrated suppressor effects that increased the predictive power of the other (Watson et al., 2013). Furthermore, cross loadings are also plausible with respect to Traumatic Avoidance and Social Anxiety on both the Distress and Obsessions/Fear Factors. Indeed, we know from trauma research that traumatic avoidance, social anxiety and depression often cooccur (e.g., Kashdan et al., 2009). Residuals of Appetite Gain and Appetite Loss factors were allowed to correlate, given their large residual value (−4.86) in our dataset, as well as in other samples (Wester et al., 2022). Additionally, these variables have a shared distress component, thus complementing each other, despite their apparently opposite meanings (Watson et al., 2013).

The CFA was conducted using the Maximum Likelihood method, with the Satorra**–**Bentler estimator, which produces standard errors robust to non-normality despite ordinal level data (Satorra and Bentler, 2001). The following indices were assessed: the Satorra-Bentler goodness-of-fit statistic χ2, the root mean square error of approximation (RMSEA), the comparative fit index (CFI), Tucker-Lewis index (TLI), and Standardized Root-Mean Residual (SRMR). Values ≥ 0.95 for TLI and CFI, <0.07 for RMSEA, and <0.08 for SRMR are generally considered to show a good model fit (Hooper et al., 2008). It should be kept in mind that a chi-square significance value < 0.05 is less meaningful in large samples (Bentler and Bonett, 1980).

With regards to missing data, participants were excluded from the analysis sample if they had missing information on the IDAS items. Eight subjects were excluded due to missing values on items 41 (“I found it difficult to make eye contact with people”) and/or 56 (“I was short of breath”). Additionally, missing scores on the MCMI scales were found for a small number of participants (10 participants for the PTSD scale, 7 for Anxiety; 6 for Thought disorder; 5 for Depressive, Dependent, Masochistic, Dysthymic, Alcohol; 4 for Schizotypal; 3 for Antisocial, Sadistic, Borderline, Drug; 2 for Negativistic; 1 for Narcissistic, Bipolar, Delusional). Pearson correlations involving these variables were handled using pairwise deletion. All other relevant variables were without missing data.

Analyses were carried out using STATA (Version 16.1).

## Results

1,064 participants completed the entire IDAS-II questionnaire, and all subsequent analyses were conducted on this sample.

### Internal consistency and unidimensionality

Table 1 shows descriptive statistics for the BDI-II, BAI, and MCMI-III.

**Table 1:** Descriptive statistics for BDI-II, BAI, and MCMI-III.

Variable	*N*	Mean	SD	Range
BDI-II	1,064	20.72	15.19	0–63
BAI	1,064	23.26	15.95	0–63
Schizoid	1,064	9.99	5.02	0–22
Avoidant	1,064	12.66	7.89	0–26
Depressive	1,059	11.45	7.66	0–23
Dependent	1,059	11.66	6.52	0–24
Histrionic	1,064	10.49	5.70	0–23
Narcissistic	1,063	12.09	5.25	0–27
Antisocial	1,061	7.53	4.31	0–23
Sadistic	1,061	11.07	5.78	0–27
Compulsive	1,064	14.00	5.18	0–25
Negativistic	1,062	13.16	6.92	0–25
Masochistic	1,059	9.16	6.76	0–22
Schizotypal	1,060	9.52	7.27	0–25
Borderline	1,061	10.00	6.68	0–25
Paranoid	1,062	9.94	6.36	0–25
Anxiety	1,057	9.50	6.19	0–20
Somatoform	1,064	7.16	4.83	0–16
Bipolar	1,063	7.00	4.26	0–18
Dysthymic	1,059	9.98	6.78	0–20
Alcohol	1,059	4.50	3.03	0–19
Drug	1,061	3.51	2.74	0–20
PTSD	1,054	8.13	6.29	0–20
Thought disorder	1,058	10.11	6.83	0–23
Major depression	1,064	10.43	7.44	0–24
Delusional	1,063	3.43	3.46	0–17

Table 2 shows means and standard deviations for scores on the IDAS-II scales, as well as average inter-item correlations (AICs), test–retest stability, the adjusted eigenvalues from the parallel analyses, and Kaiser–Meyer–Olkin (KMO) values. For most IDAS-II scales, the alpha coefficient values were higher than 0.80.

**Table 2:** Internal consistency, unidimensionality, and test–retest stability.

	M (SD)	Cronbach α	AIC	Test–retest	Adjusted eigenvalue (single factor)	KMO
General depression	61.16 (17.72)	0.93	0.40	0.84	8.92	0.95
Dysphoria	32.26 (10.68)	0.92	0.55	0.84	5.90	0.95
Lassitude	19.96 (6.09)	0.84	0.47	0.80	3.27	0.88
Insomnia	16.61 (6.11)	0.82	0.43	0.72	3.04	0.80
Suicidality	10.77 (5.89)	0.89	0.58	0.76	3.81	0.87
Appetite loss	7.99 (3.73)	0.88	0.71	52	2.36	0.73
Appetite gain	7.78 (3.52)	0.81	0.59	0.63	2.12	0.72
Well-being	22.2 (6.64)	0.84	0.39	0.74	3.80	0.90
Ill temper	14.93 (6.05)	0.91	0.66	0.79	3.51	0.88
Mania	13.78 (4.71)	0.69	0.31	0.70	2.41	0.78
Euphoria	11.03 (4.37)	0.78	0.41	0.65	2.55	0.80
Panic	21.5 (9.24)	0.92	0.58	0.82	4.95	0.92
Social anxiety	17.51 (6.85)	0.86	0.51	0.82	3.43	0.89
Claustrophobia	9.47 (5.34)	0.87	0.58	0.66	3.25	0.84
Traumatic intrusions	11.06 (4.82)	0.85	0.58	0.81	2.67	0.80
Traumatic avoidance	11.7 (4.2)	0.76	0.44	0.55	2.26	0.76
Checking	8.62 (3.45)	0.76	0.51	0.68	1.96	0.70
Ordering	12.28 (4.63)	0.71	0.33	0.63	2.24	0.65
Cleaning	14.46 (6.66)	0.87	0.48	0.72	3.87	0.90

The above results were assessed for *N* = 1,064; Test**–**retest Pearson’s correlations were carried on *N* = 154; All test–retest correlations showed large positive associations (all r > 0.52) with corresponding *p*-values < 0.001; Parallel analyses used the default seed in Stata (123456789); KMO, Kaiser–Meyer–Olkin; AIC, Average Interitem Correlations.

AIC values are generally expected to be moderate, between 0.15 and 0.50 (Clark and Watson, 1991). Most of the AIC values were moderate, except for the following scales: suicidality (0.58), Appetite Loss (0.70), Appetite Gain (0.59), Ill-Temper (0.66), Panic (0.59), and Claustrophobia (0.59), which were somewhat higher.

We used Horn’s parallel analysis and the point of inflection in scree plots to assess scale unidimensionality. All scales model only one factor. A value > 0.60 for the KMO measure was considered adequate (Kaiser, 1970) and most KMO values fit this criterion.

All scales had test–retest correlations between 0.63 (Ordering) and .84 (Dysphoria), except for Appetite Loss (0.52) and Traumatic Avoidance (0.55).

### Construct validity

Table 3 shows Pearson correlations between the IDAS-II scales, using Bonferroni correction on the calculated significance levels. Dysphoria showed the highest correlation with the other IDAS-II scales. It correlated particularly highly with Panic (0.81), Lassitude (0.78), Social Anxiety (0.77), and Traumatic Intrusions (0.75). Scales assessing specific symptoms of depression (Lassitude, Insomnia, Suicidality, Appetite Gain, Appetite Loss) were moderately associated with each other, with values ranging from *r* = 0.49 (Insomnia with Lassitude) to *r* = 0.34 (Appetite Gain with Lassitude). Scales assessing specific symptoms of anxiety (Social Anxiety, Claustrophobia, Traumatic Intrusions, Traumatic Avoidance, Ordering, Cleaning, Checking, Panic) were also moderately to strongly associated with each other, with values ranging from *r* = 0.72 (Traumatic Intrusions with Panic) to *r* = 0.25 (Cleaning with Panic).

**Table 3:** Pearson Correlations between IDAS-II subscales.

	1	2	3	4	5	6	7	8	9	10	11	12	13	14	15	16	17
1. Dysphoria	-																
2. Lassitude	0.78^***^	-															
3. Ill temper	0.68^***^	0.60^***^	-														
4. Panic	0.81^***^	0.70^***^	0.64^***^	-													
5. Traumatic intrusions	0.75^***^	0.60^***^	0.60^***^	0.72^***^	-												
6. Insomnia	0.60^***^	0.49^***^	0.46^***^	0.59^***^	0.54^***^	-											
7. Appetite loss	0.49^***^	0.45^***^	0.36^***^	0.50^***^	0.39^***^	0.44^***^	-										
8. Mania	0.56^***^	0.51^***^	0.50^***^	0.56^***^	0.54^***^	0.42^***^	0.37^***^	-									
9. Suicidality	0.60^***^	0.47^***^	0.51^***^	0.59^***^	0.59^***^	0.39^***^	0.31^***^	0.37^***^	-								
10. Traumatic Avoidance	0.26^***^	0.26^***^	0.25^***^	0.26^***^	0.31^***^	0.18^***^	0.20^***^	0.40^***^	0.13^*^	-							
11. Appetite gain	0.30^***^	0.34^***^	0.32^***^	0.25^***^	0.29^***^	0.18^***^	−0.20^***^	0.27^***^	0.21^***^	0.20^***^	-						
12. Cleaning	0.26^***^	0.26^***^	0.22^***^	0.25^***^	0.27^***^	0.18^***^	0.19^***^	0.36^***^	0.16^***^	0.30^***^	0.19^***^	-					
13. Ordering	0.29^***^	0.26^***^	0.27^***^	0.31^***^	0.32^***^	0.21^***^	0.19^***^	0.49^***^	0.14^*^	0.38^***^	0.26^***^	0.46^***^	-				
14. Checking	0.49^***^	0.45^***^	0.39^***^	0.46^***^	0.46^***^	0.33^***^	0.32^***^	0.61^***^	0.27^***^	0.43^***^	0.26^***^	0.44^***^	0.63^***^	-			
15. Claustrophobia	0.42^***^	0.36^***^	0.37^***^	0.50^***^	0.44^***^	0.35^***^	0.24^***^	0.41^***^	0.33^***^	0.28^***^	0.18^***^	0.44^***^	0.39^***^	0.43^***^	-		
16. Social anxiety	0.77^***^	0.63^***^	0.58^***^	0.70^***^	0.67^***^	0.48^***^	0.39^***^	0.50^***^	0.53^***^	0.32^***^	0.31^***^	0.32^***^	0.33^***^	0.52^***^	0.54^***^	-	
17. Euphoria	−0.21^***^	−0.13*	−0.02	−0.09	−0.08	−0.05	−0.01	0.25^***^	−0.12^*^	0.45^***^	0.12^*^	0.20^***^	0.29^***^	0.22^***^	0.10	−0.07	-
18. Wellbeing	−0.61^***^	−0.46^***^	−0.38^***^	−0.47^***^	−0.42^***^	−0.31^***^	−0.27^***^	−0.13^*^	−0.44^***^	0.11^*^	−0.06	−0.01	0.10	−0.06	−0.17^***^	−0.42^***^	0.61^***^

Pearson’s correlations between IDAS-II subscales, using Bonferroni correction on the calculated *p*-values, *N* = 1,064. Where r < 0.10 correlations were not significant. In our sample, effect sizes larger than r = 0.15 were significant at *p* < 0.001. ^***^p < 0.001; ^*^p < 0.05.

We used CFA to test Watson et al.’s (2012) three-factor structure: distress, obsessions/fear, and positive mood (see Figure 1; Watson et al., 2012). The model fit indices RMSEA = 0.073, CFI = 0.931, TLI = 0.917, SRMR = 0.056, χ2 = 840.23, *p* < 0.001, show an adequate to good model fit.

### Convergent validity

Table 4 shows Pearson correlations between the IDAS-II scale scores and the BDI-II, BAI, and MCMI-III. Correlations were conducted using Bonferroni correction on the calculated significance levels and pairwise deletion was also applied, given the missing data on MCMI-III. Correlations are displayed for the observations that have non-missing values on each pair of variables.

**Table 4:** Pearson’s correlations between the IDAS-II scale scores and BDI-II, BAI, and MCMI-III.

	1	2	3	4	5	6	7	8	9	10	11	12	13	14	15	16	17	18	19
BDI	0.83^***^	0.82^***^	0.68^***^	0.61^***^	0.73^***^	0.67^***^	0.54^***^	0.42^***^	0.49^***^	0.66^***^	0.15^***^	0.23^***^	0.22^***^	0.22^***^	0.42^***^	0.40^***^	0.67^***^	−0.27^***^	−0.62^***^
BAI	0.77^***^	0.76^***^	0.65^***^	0.62^***^	0.87^***^	0.71^***^	0.56^***^	0.47^***^	0.59^***^	0.55^***^	0.25^***^	0.25^***^	0.29^***^	0.35^***^	0.49^***^	0.52^***^	0.66^***^	−0.07	−0.43^***^
Schizoid	0.61^***^	0.59^***^	0.52^***^	0.43^***^	0.54^***^	0.51^***^	0.42^***^	0.36^***^	0.39^***^	0.46^***^	0.23^***^	0.19^***^	0.24^***^	0.24^***^	0.38^***^	0.37^***^	0.58^***^	−0.14^*^	−0.46^***^
Avoidant	0.70^***^	0.71^***^	0.58^***^	0.48^***^	0.60^***^	0.59^***^	0.43^***^	0.35^***^	0.42^***^	0.50^***^	0.24^***^	0.23^***^	0.25^***^	0.26^***^	0.46^***^	0.40^***^	0.74^***^	−0.20^***^	−0.53^***^
Depressive	0.80^***^	0.81^***^	0.67^***^	0.57^***^	0.70^***^	0.68^***^	0.51^***^	0.41^***^	0.51^***^	0.60^***^	0.24^***^	0.24^***^	0.22^***^	0.26^***^	0.46^***^	0.40^***^	0.68^***^	−0.21^***^	−0.57^***^
Dependent	0.70^***^	0.72^***^	0.59^***^	0.48^***^	0.61^***^	0.58^***^	0.41^***^	0.34^***^	0.42^***^	0.47^***^	0.22^***^	0.24^***^	0.23^***^	0.24^***^	0.43^***^	0.36^***^	0.66^***^	−0.17^***^	−0.47^***^
Histrionic	−0.55^***^	−0.54^***^	−0.42^***^	−0.34^***^	−0.46^***^	−0.43^***^	−0.32^***^	−0.28^***^	−0.26^***^	−0.44^***^	−0.14^*^	−0.13^*^	−0.2^***^	−0.14^*^	−0.30^***^	−0.33^***^	−0.61^***^	0.28^***^	0.51^***^
Narcissistic	−0.40^***^	−0.41^***^	−0.27^***^	−0.17^***^	−0.30^***^	−0.29^***^	−0.21^***^	−0.14^*^	−0.06	−0.31^***^	0.01	−0.05	−0.09	0	−0.14^*^	−0.17^***^	−0.45^***^	0.39^***^	0.46^***^
Antisocial	0.40^***^	0.41^***^	0.41^***^	0.43^***^	0.33^***^	0.34^***^	0.24^***^	0.24^***^	0.33^***^	0.31^***^	0.21^***^	0.22^***^	0.11	0.15^*^	0.21^***^	0.16_***_	0.33^***^	0.08	−0.20^***^
Sadistic	0.48^***^	0.49^***^	0.48^***^	0.58^***^	0.45^***^	0.44^***^	0.32^***^	0.29^***^	0.42^***^	0.32^***^	0.24^***^	0.27^***^	0.21^***^	0.27^***^	0.35^***^	0.28^***^	0.42^***^	0.05	−0.24^***^
Compulsive	−0.34^***^	−0.34^***^	−0.32^***^	−0.33^***^	−0.26^***^	−0.22^***^	−0.15^***^	−0.16^***^	−0.14^*^	−0.31^***^	−0.02	−0.13^*^	0.06	0.15^***^	0.04	−0.05	−0.25^***^	0.09	0.31^***^
Negativistic	0.72^***^	0.74^***^	0.65^***^	0.63^***^	0.62^***^	0.6^***^	0.45^***^	0.38^***^	0.49^***^	0.48^***^	0.27^***^	0.28^***^	0.24^***^	0.26^***^	0.44^***^	0.39^***^	0.63^***^	−0.13^*^	−0.48^***^
Masochistic	0.75^***^	0.76^***^	0.61^***^	0.54^***^	0.66^***^	0.66^***^	0.46^***^	0.38^***^	0.49^***^	0.59^***^	0.22^***^	0.25^***^	0.22^***^	0.26^***^	0.45^***^	0.43^***^	0.71^***^	−0.17^***^	−0.53^***^
Schizotypal	0.70^***^	0.70^***^	0.59^***^	0.55^***^	0.65^***^	0.64^***^	0.48^***^	0.38^***^	0.52^***^	0.59^***^	0.29^***^	0.28^***^	0.30^***^	0.33^***^	0.50^***^	0.46^***^	0.71^***^	−0.05	−0.43^***^
Borderline	0.75^***^	0.74^***^	0.64^***^	0.66^***^	0.67^***^	0.63^***^	0.45_***_	0.4^***^	0.51^***^	0.62^***^	0.24^***^	0.27^***^	0.21^***^	0.25^***^	0.40^***^	0.38^***^	0.63^***^	−0.12	−0.50^***^
Paranoid	0.58^***^	0.58_***_	0.51^***^	0.51^***^	0.54^***^	0.54^***^	0.4^***^	0.35^***^	0.49^***^	0.41^***^	0.31^***^	0.25^***^	0.30^***^	0.35^***^	0.47^***^	0.43^***^	0.59^***^	0.00	−0.32^***^
Anxiety	0.76^***^	0.77^***^	0.64^***^	0.55^***^	0.70^***^	0.74^***^	0.52^***^	0.41^***^	0.55^***^	0.52^***^	0.29^***^	0.25^***^	0.28^***^	0.34^***^	0.50^***^	0.43^***^	0.67^***^	−0.14^*^	−0.48^***^
Somatoform	0.81^***^	0.78^***^	0.73^***^	0.56^***^	0.75^***^	0.64^***^	0.59^***^	0.48^***^	0.49^***^	0.53^***^	0.2^***^	0.23^***^	0.23^***^	0.25^***^	0.43^***^	0.41^***^	0.64^***^	−0.22^***^	−0.56^***^
Bipolar	0.39^***^	0.40^***^	0.39^***^	0.40^***^	0.41^***^	0.39^***^	0.29^***^	0.27^***^	0.5^***^	0.28^***^	0.35^***^	0.26^***^	0.22^***^	0.33^***^	0.42^***^	0.29^***^	0.36^***^	0.32^***^	−0.04
Dysthymic	0.84^***^	0.84^***^	0.71^***^	0.60^***^	0.73^***^	0.68^***^	0.51^***^	0.42^***^	0.49^***^	0.6^***^	0.21^***^	0.26^***^	0.23^***^	0.24^***^	0.43^***^	0.39^***^	0.69^***^	−0.26^***^	−0.62^***^
Alcohol	0.46^***^	0.46^***^	0.43^***^	0.44^***^	0.39^***^	0.37^***^	0.28^***^	0.27^***^	0.35^***^	0.37^***^	0.19^***^	0.24^***^	0.14^*^	0.18^***^	0.27^***^	0.21^***^	0.37^***^	0.03	−0.25^***^
Drug	0.24^***^	0.23^***^	0.26^***^	0.28^***^	0.18^***^	0.19^***^	0.11	0.14^*^	0.18^***^	0.22^***^	0.11	0.17^***^	0.05	0.07	0.09	0.07	0.16^***^	0.07	−0.09
PTSD	0.71^***^	0.7^***^	0.59^***^	0.55^***^	0.65^***^	0.76^***^	0.49^***^	0.37^***^	0.52^***^	0.57^***^	0.28^***^	0.24^***^	0.25^***^	0.30^***^	0.45^***^	0.40^***^	0.61^***^	−0.14^*^	−0.46^***^
Thought disorder	0.79^***^	0.79^***^	0.66^***^	0.62^***^	0.72^***^	0.68^***^	0.51^***^	0.45^***^	0.56^***^	0.60^***^	0.27^***^	0.27^***^	0.27^***^	0.30^***^	0.48^***^	0.44^***^	0.68^***^	−0.12^*^	−0.51^***^
Major depression	0.85^***^	0.81^***^	0.72^***^	0.59^***^	0.74^***^	0.67^***^	0.59^***^	0.49^***^	0.48^***^	0.63^***^	0.17^***^	0.21^***^	0.20^***^	0.20^***^	0.40^***^	0.38^***^	0.65^***^	−0.27^***^	−0.61^***^
Delusional	0.38^***^	0.38^***^	0.34^***^	0.38^***^	0.41^***^	0.38^***^	0.27^***^	0.25^***^	0.4^***^	0.34^***^	0.25^***^	0.19^***^	0.20^***^	0.27^***^	0.33^***^	0.32^***^	0.38^***^	0.15^*^	−0.13^*^

Correlations depict results for non-missing variables pairs (*N* = 1,054 for PTSD; *N* = 1,057 for Anxiety; *N* = 1,058 for Thought disorder; *N* = 1,059 for Depressive, Dependent, Masochistic, Dysthymic, Alcohol; *N* = 1,060 for Schizotypal; *N* = 1,061 for Antisocial, Sadistic, Borderline, Drug; *N* = 1,062 for Negativistic; *N* = 1,063 for Narcissistic, Bipolar, Delusional; *N* = 1,064 for all other correlations); Where r < 0.11 correlations were not significant. In our sample, effect sizes larger than r = 0.15 were significant at *p* < 0.001; 1. General Depression; 2. Dysphoria; 3. Lassitude; 4. Ill Temper; 5. Panic; 6. Traumatic Intrusions; 7. Insomnia; 8. Appetite Loss; 9. Mania; 10. Suicidality; 11. Traumatic Avoidance; 12. Appetite Gain; 13. Cleaning; 14. Ordering; 15. Checking; 16. Claustrophobia; 17. Social Anxiety; 18. Euphoria; 19. Well-Being. ^***^p < 0.001; ^*^p < 0.05.

Scales assessing the same constructs showed the highest correlation. For example, the highest correlation was observed between the IDAS-II General Depression scale and the MCMI-III Major Depression scale (0.85), the BDI-II (0.83), and the MCMI-III Dysthymia scale (0.83). This pattern was similar for all scales assessing depressive symptomatology.

Among the IDAS-II scales assessing anxiety symptoms, the highest correlations were between the Panic scale and the BAI (0.87). The highest association of the Traumatic Intrusions scale was with the MCMI-III PTSD scale (0.76). The Social Anxiety scale demonstrated large associations with the MCMI-III Dysthymia scale (0.69), MCMI-III Thought Disorder scale (0.68) and MCMI-III Anxiety Scale (0.67). The Mania scale showed a moderate positive association with MCMI-III Bipolar scale (0.32). The Well-Being scale exhibited large negative associations with the MCMI-III Dysthymia scale and the BDI-II total score (both *r*s = −0.62). The IDAS-II Euphoria scale showed moderate positive association with the MCMI-III Narcissistic PD scale (0.39).

With regards to the MCMI-III’s personality disorder scales, all correlations were found to be significant, except for those between Histrionic PD and Appetite Gain, and Ordering; Narcissistic PD and Appetite Loss, and Checking; Compulsive PD and Mania, and Checking; and Antisocial PD and Euphoria. The IDAS-II scales and the MCMI-III Histrionic and Narcissistic PD scales were negatively associated, except for those involving the Ill Temper, Panic, Euphoria, and the Well-Being scales, which were positive. The associations between the IDAS-II scales and the MCMI-III Compulsive PD scale were negative, except for those with Ill Temper, Panic, Euphoria, and Well-Being. The MCMI-III Compulsive PD scale also demonstrated moderate/large positive associations with the Cleaning, Ordering, and Checking scales.

The MCMI-III PD scales and the IDAS-II scales showed the following moderate/large positive associations: the IDAS-II General Depression scale and the MCMI-III Depressive (0.80) and Borderline scales (0.75); the IDAS-II Dysphoria and Lassitude scales and the MCMI-III Depressive (0.81, 0.67) and Borderline scales (0.74, 0.64); the IDAS-II Ill Temper scale and the MCMI-III Borderline (0.66) and Negativistic scales (0.63); the IDAS-II Panic scale and the MCMI-III Depressive (0.70) and Borderline scales (0.67); the IDAS-II Traumatic Intrusions scale and the MCMI-III Depressive (0.68) and Masochistic scales (0.68); the IDAS-II Insomnia scale and the MCMI-III Depressive (0.51) and Schizotypal scales (0.48); the IDAS-II Appetite Scale and the MCMI-III Depressive (0.41) and Borderline scales (0.40); the IDAS-II Mania scale and the MCMI-III Schizotypal (0.51), Depressive (0.51) and Borderline scales (0.51); the IDAS-II Suicidality scale and the MCMI-III Borderline (0.62), Depressive (0.60), and Schizotypal scales (0.59); the IDAS-II Traumatic Avoidance scale and the MCMI-III Paranoid (0.39) and Schizotypal scales (0.29); the IDAS-II Cleaning scale and the MCMI-III Paranoid (0.30) and Schizotypal scales (0.30); the IDAS-II Ordering scale and the MCMI-III Paranoid (0.35) and Schizotypal scales (0.33); the IDAS-II Checking scale and the MCMI-III Schizotypal (0.50) and Paranoid scales (0.47); the IDAS-II Claustrophobia scale and the MCMI-III Schizotypal (0.46), Paranoid (0.43), and Masochistic scales (0.43); the IDAS-II Social Anxiety scale and the MCMI-III Depressive (0.74) and Schizotypal scales (0.71); the IDAS-II Euphoria scale and the MCMI-III Narcissistic scale (0.39). To this end, the IDAS-II Well-Being scale showed moderate positive associations with the MCMI-III Narcissistic PD (0.46) and Histrionic PD scales (0.51).

One significant limitation of these findings is that they are based on a sample that is predominantly (89.9%) female. Consequently, we provide a separate analysis on a subsample of gender-balanced participants, which we include in Supplementary Tables S1–S5. Specifically, the analysis was rerun on *N* = 212 (106 males together with a random subsample of 106 females; females were selected at random using a seed, for reproducibility: 36548292), and the results were in line with those from the overall sample. We note, however, the reduced predictive power in this smaller sample. Comparisons between men and women (*N* = 212; 106 males, 106 females), using the Mann–Whitney *U*-test, showed statistically significant differences at Bonferroni corrected α < 0.003 in all the scores of the IDAS-II scales, except for Checking, Ordering, Appetite Gain, Cleaning, Traumatic Avoidance, Suicidality, and Mania (Supplementary Table S5). Women scored higher on all scales, except for IDAS-II Well-Being.

We also note a further limitation related to the age distribution in our sample. Therefore, an additional age-related analysis was conducted on *N* = 116 based on the following selection: all available participants aged 45–65 (58 participants), together with an equal subsample of randomly selected participants aged 19–44 (seed for random selection: 36548292). Between-group comparisons were conducted using the Mann–Whitney *U*-test (Supplementary Table S6). The results showed statistically significant differences between age groups for all the scores of the IDAS-II scales, except for Euphoria, Claustrophobia, Ordering, and Cleaning. Participants aged 19–44 scored higher on all scales except Well-Being. Furthermore, a CFA conducted on all participants aged 19–44 (*N* = 1,006) yielded results similar to those identified in the full sample (Supplementary Table S7).

Finally, a supplemental sensitivity check was conducted using one-way analyses of variance, separately on each IDAS-II scale between mild, moderate, and severe groupings on the BDI and BAI measures (*N* = 1.064). All comparisons except Euphoria (BAI groups) were significant at the adjusted *α* (all *p* < 0.001; Supplementary Table S8).

## Discussion

This study provides evidence for the reliability and validity of the Romanian version of the IDAS-II. Previous research using English, Turkish, Spanish, Swedish, and German versions yielded comparable effects (Watson et al., 2012; de la Rosa-Cáceres et al., 2020; Irak and Albayrak, 2020; Wester et al., 2022; Cervin et al., 2023). The findings are consistent with both the hypothesized internal structure of the IDAS-II and the HiTOP model (Kotov et al., 2017). Thus, congruent with previous IDAS-II studies (Watson et al., 2012; de la Rosa-Cáceres et al., 2020; Irak and Albayrak, 2020; Wester et al., 2022; Cervin et al., 2023), the Cronbach alpha values exceeded 0.80 for most of the IDAS-II scales, showing an appropriate level of internal consistency. The General Depression scale demonstrated the highest internal consistency. Similar to previous adaptations of the IDAS-II, the Traumatic Avoidance, Mania, and Euphoria scales showed the lowest internal consistency values (Watson et al., 2012; de la Rosa-Cáceres et al., 2020; Irak and Albayrak, 2020; Wester et al., 2022; Cervin et al., 2023).

The test–retest correlations in the seven-to-10-day interval were large in magnitude for most scales (*N* = 154). However, the Traumatic Avoidance, Appetite Loss, Appetite Gain scales exhibited lower test**–**retest associations, albeit still moderately/highly positive. Consistent with the findings of the IDAS-II authors, these results may be explained by symptoms’ susceptibility to change (Watson et al., 2012; Watson and O’Hara, 2017).

In congruence with the instrument’s operational design and the theoretical HiTOP model, the Romanian version of the IDAS-II exhibits small differences regarding scale intercorrelations, as compared to the original IDAS-II (Kotov et al., 2017, 2021). Strong associations between scales belonging to the same factor indicate they assess distinct, but related variables. Lower correlations between measures purported to assess different disorders were found, demonstrating that this instrument is capable of tapping into specific symptoms linked with each disorder. Findings of the current study are consistent with those of earlier investigations conducted to validate the IDAS-II (Watson et al., 2012; de la Rosa-Cáceres et al., 2020; Irak and Albayrak, 2020; Wester et al., 2022). Despite lower fit values of the CFA, it can be inferred that the internal structure of the Romanian version of the IDAS-II is largely consistent with the original form, given the high internal consistency discussed above.

CFA results indicated that the original three-factor model of the Romanian version of IDAS-II—consisting of Distress, Obsessions/Fear, and Positive Mood—showed an acceptable statistical fit.

In terms of the convergent and discriminant validity of the IDAS-II scales, results showed moderate-to-strong associations of General Depression and Dysphoria with other scales that measure the same constructs, highlighting the potential of these scales as screening instruments for internalizing disorders (see also Stasik-O’Brien et al., 2019). Moreover, the specific symptom scales showed strong associations with corresponding instruments and lower correlations with non-corresponding measures. More specifically, the IDAS-II General Depression scale showed the strongest positive associations with the MCMI-III Major Depression scale, the BDI-II total score, and the MCMI-III Dysthymia scale; whereas the Panic scale demonstrated the strongest positive correlations with the BAI, the Social Anxiety scale with the MCMI-III Dysthymia and Anxiety scales, the Mania scale with the MCMI-III Bipolar scale, and the Traumatic Intrusions scale with the MCMI-III PTSD scale. The Well-Being scale exhibited the strongest negative associations with the MCMI-III Dysthymia scale and BDI-II total score.

In line with results reported by Watson and O’Hara (2017), which indicated that the IDAS-II Euphoria scale assesses a dysfunctional form of positive affect, our findings confirm that this scale is positively associated with the MCMI-III Histrionic PD and the MCMI-III Narcissistic PD scales, although these correlations were relatively weak. Interestingly, the IDAS-II Well-Being scale had somewhat stronger positive associations with the MCMI-III Histrionic and Narcissistic PD scales.

Large positive associations were found between the IDAS-II General Depression, Dysphoria, Lassitude, Ill Temper, Panic, Appetite Loss, Mania, and Suicidality scales on the one hand, and the MCMI-III Borderline scale on the other. These results are congruent with those obtained by Anderson et al. (2018) which showed that borderline personality, as measured using the Personality Diagnostic Questionnaire-4 (PDQ-4), was associated with, and predicted by all theoretically expected PID-5-BF (Personality Inventory for DSM-5–Brief Form) domains (Krueger et al., 2012). PID-5-BF-Negative affectivity was found to be the strongest predictor of borderline personality, and of the IDAS-II scales, General Depression and Dysphoria (Anderson et al., 2018).

Some of the strongest associations observed were between the MCMI-III Schizotypal PD and Paranoid PD scales on the one hand, and the IDAS-II Cleaning, Ordering, Checking, and Claustrophobia scales on the other. The associations between the Schizotypal PD and the IDAS-II OCD scales are in line with previous results indicating a strong relationship between OCD and schizotypal PD (Rossi and Derksen, 2015). Furthermore, PID-5-BF Psychoticism was correlated with each PD measured by PDQ-4 (Anderson et al., 2018), Schizotypal PD being a stronger predictor of PID-5 Psychoticism than Avoidance, Borderline, Antisocial, Narcissistic, and Obsessive–Compulsive PDs (Paranoid PD was not measured). Nonetheless, as shown by Anderson et al. (2018) and Watson and O’Hara (2017), PID-5 Psychoticism displayed strong links with the IDAS-II OCD scales (Cleaning, Ordering, Checking) and the two bipolar scales (Mania, Euphoria). We found similar results, except for the IDAS-II Euphoria scale, which was not associated with the MCMI-III Schizotypal PD or Paranoid PD scales.

The MCMI-III Somatoform scale was strongly associated with IDAS-II General Depression. Moreover, the MCMI-III Somatoform scale correlated moderately or highly with the Dysphoria, Lassitude, Insomnia, Suicidality, Appetite Loss, Ill Temper, Mania, Panic, Social Anxiety, Claustrophobia, Traumatic Intrusions, and Checking scales, and slightly with Appetite Gain, Ordering and Cleaning, and Traumatic Avoidance. The MCMI-III Somatoform scale had also a low negative association with the Euphoria scale, and a moderate negative association with the Well-Being scale. These results are important, considering extant literature regarding the capacity of the IDAS-II to measure symptoms related to hypochondriasis, termed *illness anxiety disorder* in DSM-5. In a previous study (Watson and O’Hara, 2017), Hypochondriacal Alienation and Hypochondriacal Worry measured with the Multidimensional Inventory of Hypochondriacal Traits (MIHT; Longley et al., 2005) both displayed a moderate overlap with the IDAS-II, as opposed to the Hypochondriacal Reassurance and Hypochondriacal Absorption scales, which were weakly related to the IDAS-II. In this respect, the DSM-IV Somatoform disorders were moved to a new DSM-5 category called Somatic Symptoms and Related Disorders that includes diagnoses of Somatic Symptom Disorder, Illness Anxiety Disorder, Conversion Disorder, Factitious Disorder, and various other related conditions. The results of this study show that the IDAS-II contains the appropriate content to capture important variations in the Somatic Symptoms spectrum.

The Euphoria and Well-Being scales, which represent the third factor of the IDAS-II called Positive Mood, showed a significant association with the MCMI-III Histrionic PD and the MCMI-III Narcissistic PD scales. These results could be explained by the fact that Histrionic Personality Disorder is considered to implicate more than one facet of extraversion, including positive emotionality (Lynam and Widiger, 2001).

Given the preponderance of women in our sample, this might have an influence on depression and anxiety dimensions. Although the smaller sample for the additional analyses on a gender-balanced sub-samples limits the interpretation of the results, we note that they are suggestive of the fact that the Romanian version of the IDAS-II could capture expected differences in age and gender, based on previously published norms (Sanchez-Garcia et al., 2021).

### Limitations and future directions

Although this study provides evidence establishing both the reliability and validity of the scales of the IDAS-II-Romanian Version, some limitations are worth noting. First, this study was conducted exclusively on a community sample. Therefore, future studies should investigate the psychometric properties of the IDAS-II Romanian version in more heterogeneous samples, including patients with psychiatric conditions. Second, the sample was mainly composed of young women and students. Even though results from the sub-sample of gender-balanced participants are in line with those from the study’s overall sample, it should be considered that their predictive power is smaller. Future studies should be conducted on representative samples of men, middle-aged adults, and older adults. Third, convergent and discriminant validity were investigated only for a subset of the IDAS-II scales and were limited by the use of self-report measures. This study was unable to employ gold-standard measures that specifically evaluate Well-Being, OCD, and Claustrophobia. Future studies should include instruments that evaluate these symptoms, to test the convergent and discriminant validity of such scales.

## Conclusion

This is the first study to adapt and validate the IDAS-II to the Romanian population. Moreover, this study provides additional evidence for the correspondence between the internal structure of the IDAS-II and the hierarchical taxonomy of the HiTOP model. These data leave the door open for continued research on the symptoms that comprise the HiTOP model’s Internalizing spectrum. Considering the outcomes reached and the advantages of this tool, the IDAS-II confirms its potential in assessing the severity of depression, anxiety, and bipolar symptoms for the Romanian population in scientific and clinical transdiagnostic contexts. Future research should be performed to validate this instrument’s psychometric characteristics in Romanian subpopulations, namely, patients and adolescents, and to prove its value in clinical settings.

## Data availability statement

The original contributions presented in the study are included in the article/Supplementary material, further inquiries can be directed to the corresponding author.

## Ethics statement

This study was approved by the Research Ethics Committee of the University of Bucharest (IRB no 10/25.01.2022). The participants provided their written informed consent to participate in this study.

## Author contributions

LMP: conceptualization, methodology, data analysis and interpretation, and writing—original draft, review and editing. DAG: data analysis and interpretation and writing—review and editing. DW: conceptualization, methodology, and writing—review and editing. LM: data acquisition and writing—review and editing. All authors contributed to the article and approved the submitted version.

## Funding

DAG was supported by a grant from the Ministry of Research, Innovation and Digitization, CNCS—UEFISCDI, project number PN-III-P1-1.1-PD-2021-0138, within PNCDI III.

## Conflict of interest

The authors declare that the research was conducted in the absence of any commercial or financial relationships that could be construed as a potential conflict of interest.

## Publisher’s note

All claims expressed in this article are solely those of the authors and do not necessarily represent those of their affiliated organizations, or those of the publisher, the editors and the reviewers. Any product that may be evaluated in this article, or claim that may be made by its manufacturer, is not guaranteed or endorsed by the publisher.
