# Supplementary material for: Romanian Inventory of Depression and Anxiety Symptoms (IDAS-II)
Source: Front Psychol. 2023 Jul 6;14:1159380. doi: 10.3389/fpsyg.2023.1159380 (PMC10359186; doi:10.3389/fpsyg.2023.1159380)
Supplement: Supplementary file 1 [file Data_Sheet_1.docx]

**Romanian Adaptation of the Inventory of Depression and Anxiety Symptoms (IDAS-II)**

**Supplementary Results**

Table S1.

Internal Consistency, Unidimentionality, and Test**–**Retest Stability for Male participants together with an equal subsample of Females

|  | M (SD) | Cronbach α | AIC | Test – retest | Adjusted eigenvalue (single factor) | KMO |
| --- | --- | --- | --- | --- | --- | --- |
| General Depression | 59.38 (18.45) | 0.93 | 0.42 | 0.87 | 8.85 | 0.91 |
| Dysphoria | 30.95 (10.68) | 0.92 | 0.53 | 0.86 | 5.56 | 0.93 |
| Lassitude | 19.26 (6.31) | 0.85 | 0.48 | 0.85 | 3.24 | 0.85 |
| Insomnia | 16.63 (6.06) | 0.80 | 0.41 | 0.85 | 2.85 | 0.76 |
| Suicidality | 10.75 (6.08) | 0.91 | 0.62 | 0.86 | 3.90 | 0.86 |
| Appetite Loss | 7.71 (3.85) | 0.89 | 0.73 | 0.68 | 2.36 | 0.73 |
| Appetite Gain | 7.62 (3.44) | 0.78 | 0.54 | 0.64 | 1.99 | 0.63 |
| Well-Being | 22.72 (6.74) | 0.84 | 0.39 | 0.83 | 3.68 | 0.88 |
| Ill Temper | 14.39 (5.93) | 0.90 | 0.65 | 0.85 | 3.41 | 0.85 |
| Mania | 13.64 (4.69) | 0.70 | 0.32 | 0.69 | 2.38 | 0.79 |
| Euphoria | 11.39 (4.34) | 0.75 | 0.37 | 0.68 | 2.33 | 0.77 |
| Panic | 20.25 (9.63) | 0.93 | 0.62 | 0.85 | 5.09 | 0.91 |
| Social Anxiety | 16.59 (6.88) | 0.86 | 0.50 | 0.80 | 3.33 | 0.90 |
| Claustrophobia | 8.5 (4.81) | 0.86 | 0.55 | 0.51 | 3.12 | 0.88 |
| Traumatic Intrusions | 10.93 (4.9) | 0.85 | 0.59 | 0.86 | 2.61 | 0.79 |
| Traumatic Avoidance | 11.84 (4.34) | 0.77 | 0.46 | 0.59 | 2.20 | 0.78 |
| Checking | 8.71 (3.39) | 0.74 | 0.49 | 0.67 | 1.89 | 0.67 |
| Ordering | 12.43 (4.73) | 0.72 | 0.34 | 0.70 | 2.22 | 0.65 |
| Cleaning | 14.32 (7.04) | 0.88 | 0.52 | 0.62 | 3.84 | 0.90 |

***Note.*** The above results were assessed for *N* = 212; Test**–**Retest Pearson Correlations were carried on *N* = 43 (17 Females; 26 Males); All test-retest correlations showed large positive associations (all *r* > .51) with corresponding *p-*values < .001; Parallel analyses used the default seed in Stata (123456789); KMO = Kaiser– Meyer–Olkin; AIC = Average Interitem Correlations

Table S2.

Pearson Correlations between IDAS-II Subscales for Male participants together with an equal subsample of Females

|  | 1 | 2 | 3 | 4 | 5 | 6 | 7 | 8 | 9 | 10 | 11 | 12 | 13 | 14 | 15 | 16 | 17 |
| --- | --- | --- | --- | --- | --- | --- | --- | --- | --- | --- | --- | --- | --- | --- | --- | --- | --- |
| 1. Dysphoria | - |  |  |  |  |  |  |  |  |  |  |  |  |  |  |  |  |
| 2. Lassitude | 0.81*** | - |  |  |  |  |  |  |  |  |  |  |  |  |  |  |  |
| 3. Ill Temper | 0.64*** | 0.59*** | - |  |  |  |  |  |  |  |  |  |  |  |  |  |  |
| 4. Panic | 0.79*** | 0.71*** | 0.65*** | - |  |  |  |  |  |  |  |  |  |  |  |  |  |
| 5. Traumatic Intrusions | 0.79*** | 0.69*** | 0.56*** | 0.78*** | - |  |  |  |  |  |  |  |  |  |  |  |  |
| 6. Insomnia | 0.69*** | 0.59*** | 0.55*** | 0.62*** | 0.62*** | - |  |  |  |  |  |  |  |  |  |  |  |
| 7. Appetite Loss | 0.54*** | 0.49*** | 0.45*** | 0.56*** | 0.52*** | 0.54*** | - |  |  |  |  |  |  |  |  |  |  |
| 8. Mania | 0.58*** | 0.52*** | 0.47*** | 0.56*** | 0.53*** | 0.4*** | 0.37*** | - |  |  |  |  |  |  |  |  |  |
| 9. Suicidality | 0.59*** | 0.48*** | 0.49*** | 0.61*** | 0.61*** | 0.44*** | 0.47*** | 0.42*** | - |  |  |  |  |  |  |  |  |
| 10. Traumatic Avoidance | 0.44*** | 0.36*** | 0.35*** | 0.46*** | 0.47*** | 0.31*** | 0.3* | 0.44*** | 0.22 | - |  |  |  |  |  |  |  |
| 11. Appetite Gain | 0.25* | 0.33*** | 0.26* | 0.22 | 0.22 | 0.12 | -0.23 | 0.3* | 0.13 | 0.17 | - |  |  |  |  |  |  |
| 12. Cleaning | 0.37*** | 0.34*** | 0.23* | 0.31*** | 0.37*** | 0.26* | 0.25* | 0.35*** | 0.19 | 0.37*** | 0.14 | - |  |  |  |  |  |
| 13. Ordering | 0.32*** | 0.29* | 0.26* | 0.3* | 0.32*** | 0.20 | 0.20 | 0.52*** | 0.12 | 0.41*** | 0.25* | 0.43*** | - |  |  |  |  |
| 14. Checking | 0.47*** | 0.44*** | 0.33*** | 0.45*** | 0.46*** | 0.32*** | 0.28* | 0.6*** | 0.25* | 0.51*** | 0.3* | 0.44*** | 0.69*** | - |  |  |  |
| 15. Claustrophobia | 0.47*** | 0.41*** | 0.41*** | 0.52*** | 0.47*** | 0.37*** | 0.36*** | 0.48*** | 0.3*** | 0.35*** | 0.22 | 0.59*** | 0.46*** | 0.51*** | - |  |  |
| 16. Social Anxiety | 0.76*** | 0.65*** | 0.55*** | 0.69*** | 0.73*** | 0.63*** | 0.54*** | 0.45*** | 0.52*** | 0.42*** | 0.24 | 0.41*** | 0.34*** | 0.53*** | 0.56*** | - |  |
| 17. Euphoria | -0.21 | -0.14 | -0.10 | -0.11 | -0.09 | -0.10 | -0.12 | 0.26* | -0.11 | 0.21 | 0.18 | 0.10 | 0.27* | 0.24 | 0.06 | -0.08 | - |
| 18. Well-Being | -0.62*** | -0.47*** | -0.4*** | -0.5*** | -0.47*** | -0.46*** | -0.36*** | -0.23 | -0.41*** | -0.12 | 0.02 | -0.12 | 0.05 | -0.04 | -0.25* | -0.4*** | 0.59*** |

***Note.*** Pearson correlations between IDAS-II subscales, using Bonferroni correction on the calculated *p* values; *N* = 212; *** *p* < .001; * *p* < .05

Table S3.

Pearson Correlations between the IDAS-II Scale Scores and BDI-II, BAI, and MCMI-III for Male participants together with an equal subsample of Females.

|  | 1 | 2 | 3 | 4 | 5 | 6 | 7 | 8 | 9 | 10 | 11 | 12 | 13 | 14 | 15 | 16 | 17 | 18 | 19 |
| --- | --- | --- | --- | --- | --- | --- | --- | --- | --- | --- | --- | --- | --- | --- | --- | --- | --- | --- | --- |
| BDI-II | 0.84*** | 0.81*** | 0.69*** | 0.63*** | 0.76*** | 0.73*** | 0.62*** | 0.55*** | 0.56*** | 0.67*** | 0.35*** | 0.18 | 0.27 | 0.25 | 0.42*** | 0.44*** | 0.68*** | -0.27 | -0.62*** |
| BAI | 0.76*** | 0.75*** | 0.7*** | 0.62*** | 0.88*** | 0.75*** | 0.56*** | 0.56*** | 0.58*** | 0.57*** | 0.46*** | 0.23 | 0.35*** | 0.41*** | 0.52*** | 0.55*** | 0.69*** | -0.12 | -0.49*** |
| Schizoid | 0.66*** | 0.65*** | 0.57*** | 0.47*** | 0.62*** | 0.59*** | 0.5*** | 0.44*** | 0.49*** | 0.51*** | 0.4*** | 0.14 | 0.3* | 0.26 | 0.44*** | 0.42*** | 0.64*** | -0.09 | -0.53*** |
| Avoidant | 0.69*** | 0.68*** | 0.56*** | 0.46*** | 0.61*** | 0.61*** | 0.54*** | 0.41*** | 0.48*** | 0.5*** | 0.41*** | 0.17 | 0.34*** | 0.33* | 0.51*** | 0.52*** | 0.75*** | -0.14 | -0.52*** |
| Depressive | 0.82*** | 0.8*** | 0.71*** | 0.59*** | 0.71*** | 0.71*** | 0.61*** | 0.53*** | 0.6*** | 0.61*** | 0.38*** | 0.19 | 0.3* | 0.31* | 0.45*** | 0.49*** | 0.66*** | -0.19 | -0.62*** |
| Dependent | 0.66*** | 0.68*** | 0.62*** | 0.4*** | 0.6*** | 0.59*** | 0.45*** | 0.39*** | 0.44*** | 0.42*** | 0.36*** | 0.13 | 0.33*** | 0.28* | 0.39*** | 0.44*** | 0.62*** | -0.17 | -0.48*** |
| Histrionic | -0.59*** | -0.56*** | -0.45*** | -0.38*** | -0.5*** | -0.49*** | -0.49*** | -0.37*** | -0.35*** | -0.44*** | -0.34*** | -0.08 | -0.3* | -0.14 | -0.37*** | -0.36*** | -0.62*** | 0.22 | 0.55*** |
| Narcissistic | -0.38*** | -0.4*** | -0.27 | -0.17 | -0.29* | -0.27 | -0.3* | -0.16 | -0.09 | -0.2 | -0.13 | 0.03 | -0.19 | 0.02 | -0.13 | -0.22 | -0.44*** | 0.39*** | 0.49*** |
| Antisocial | 0.42*** | 0.45*** | 0.45*** | 0.41*** | 0.36*** | 0.4*** | 0.37*** | 0.27 | 0.37*** | 0.33* | 0.23 | 0.29* | 0.17 | 0.22 | 0.24 | 0.26 | 0.32* | 0.15 | -0.21 |
| Sadistic | 0.52*** | 0.51*** | 0.49*** | 0.6*** | 0.51*** | 0.56*** | 0.47*** | 0.36*** | 0.41*** | 0.41*** | 0.31* | 0.23 | 0.24 | 0.25 | 0.33* | 0.34*** | 0.46*** | 0.01 | -0.31* |
| Compulsive | -0.43*** | -0.44*** | -0.41*** | -0.37*** | -0.34*** | -0.31* | -0.34*** | -0.27 | -0.18 | -0.32* | -0.05 | -0.17 | -0.09 | 0.09 | -0.03 | -0.1 | -0.29* | 0.05 | 0.32* |
| Negativistic | 0.74*** | 0.75*** | 0.67*** | 0.61*** | 0.67*** | 0.68*** | 0.57*** | 0.46*** | 0.53*** | 0.53*** | 0.41*** | 0.27 | 0.33*** | 0.3* | 0.44*** | 0.46*** | 0.63*** | -0.09 | -0.49*** |
| Masochistic | 0.73*** | 0.73*** | 0.59*** | 0.52*** | 0.68*** | 0.66*** | 0.53*** | 0.49*** | 0.56*** | 0.59*** | 0.38*** | 0.14 | 0.31* | 0.33*** | 0.48*** | 0.53*** | 0.69*** | -0.11 | -0.53*** |
| Schizotypal | 0.7*** | 0.7*** | 0.58*** | 0.5*** | 0.66*** | 0.67*** | 0.55*** | 0.46*** | 0.52*** | 0.6*** | 0.4*** | 0.23 | 0.38*** | 0.38*** | 0.56*** | 0.56*** | 0.74*** | -0.03 | -0.43*** |
| Borderline | 0.76*** | 0.75*** | 0.67*** | 0.66*** | 0.69*** | 0.69*** | 0.54*** | 0.51*** | 0.54*** | 0.65*** | 0.38*** | 0.22 | 0.26 | 0.25 | 0.39*** | 0.44*** | 0.62*** | -0.07 | -0.52*** |
| Paranoid | 0.59*** | 0.58*** | 0.49*** | 0.46*** | 0.58*** | 0.58*** | 0.45*** | 0.45*** | 0.5*** | 0.49*** | 0.43*** | 0.21 | 0.32* | 0.4*** | 0.51*** | 0.49*** | 0.61*** | 0.01 | -0.35*** |
| Anxiety | 0.75*** | 0.76*** | 0.68*** | 0.54*** | 0.71*** | 0.76*** | 0.54*** | 0.51*** | 0.53*** | 0.53*** | 0.47*** | 0.2 | 0.38*** | 0.4*** | 0.52*** | 0.5*** | 0.72*** | -0.11 | -0.47*** |
| Somatoform | 0.84*** | 0.81*** | 0.77*** | 0.6*** | 0.79*** | 0.71*** | 0.63*** | 0.58*** | 0.52*** | 0.55*** | 0.4*** | 0.17 | 0.29* | 0.29* | 0.44*** | 0.46*** | 0.69*** | -0.23 | -0.61*** |
| Bipolar | 0.41*** | 0.45*** | 0.45*** | 0.35*** | 0.46*** | 0.51*** | 0.3* | 0.33* | 0.5*** | 0.35*** | 0.36*** | 0.23 | 0.25 | 0.36*** | 0.42*** | 0.36*** | 0.39*** | 0.34*** | -0.06 |
| Dysthymic | 0.86*** | 0.86*** | 0.74*** | 0.61*** | 0.76*** | 0.74*** | 0.61*** | 0.51*** | 0.57*** | 0.61*** | 0.41*** | 0.18 | 0.33*** | 0.27 | 0.45*** | 0.49*** | 0.69*** | -0.21 | -0.64*** |
| Alcohol | 0.48*** | 0.5*** | 0.48*** | 0.41*** | 0.43*** | 0.45*** | 0.38*** | 0.3* | 0.35*** | 0.37*** | 0.29* | 0.23 | 0.23 | 0.26 | 0.3* | 0.32* | 0.4*** | 0.06 | -0.31* |
| Drug | 0.2 | 0.21 | 0.24 | 0.22 | 0.12 | 0.21 | 0.17 | 0.16 | 0.2 | 0.17 | 0.07 | 0.22 | 0.07 | 0.11 | 0.07 | 0.14 | 0.15 | 0.18 | 0 |
| PTSD | 0.75*** | 0.73*** | 0.66*** | 0.57*** | 0.72*** | 0.77*** | 0.56*** | 0.51*** | 0.55*** | 0.59*** | 0.4*** | 0.21 | 0.31* | 0.32* | 0.46*** | 0.46*** | 0.66*** | -0.1 | -0.47*** |
| Thought Disorder | 0.8*** | 0.8*** | 0.69*** | 0.6*** | 0.74*** | 0.74*** | 0.59*** | 0.55*** | 0.57*** | 0.63*** | 0.39*** | 0.19 | 0.37*** | 0.32* | 0.48*** | 0.51*** | 0.71*** | -0.1 | -0.55*** |
| Major Depression | 0.88*** | 0.83*** | 0.75*** | 0.63*** | 0.77*** | 0.74*** | 0.65*** | 0.58*** | 0.52*** | 0.67*** | 0.37*** | 0.15 | 0.28* | 0.24 | 0.4*** | 0.43*** | 0.68*** | -0.25 | -0.64*** |
| Delusional | 0.43*** | 0.41*** | 0.33*** | 0.38*** | 0.4*** | 0.41*** | 0.32* | 0.36*** | 0.38*** | 0.41*** | 0.28* | 0.17 | 0.18 | 0.29* | 0.36*** | 0.36*** | 0.42*** | 0.11 | -0.18 |

***Note.*** The above results were assessed for N = 212; 1. General Depression; 2. Dysphoria; 3. Lassitude; 4. Ill Temper; 5. Panic; 6. Traumatic Intrusions; 7. Insomnia; 8. Appetite Loss; 9. Mania; 10. Suicidality; 11. Traumatic Avoidance; 12. Appetite Gain; 13. Cleaning; 14. Ordering; 15. Checking; 16. Claustrophobia; 17. Social Anxiety; 18. Euphoria; 19. Well-Being; ; *N* = 212; *** *p* < .001; * *p* < .05

Table S4.

Confirmatory Factor Analysis for Male participants together with an equal subsample of Females

|  | Distress | Obsessions/Fear | Positive Mood | |
| --- | --- | --- | --- | --- |
| Dysphoria | 0.93 |  |  |  |
| Lassitude | 0.83 |  |  |  |
| Ill Temper | 0.71 |  |  |  |
| Panic | 0.87 |  |  |  |
| Traumatic Intrusions | 0.86 |  |  |  |
| Insomnia | 0.73 |  |  |  |
| Appetite Loss | 0.62 |  |  |  |
| Suicidality | 0.65 |  |  |  |
| Appetite Gain | 0.27 |  |  |  |
| Mania | 0.70 |  | 0.40 |  |
| Social Anxiety | 0.69 | 0.19 |  |  |
| Traumatic Avoidance | 0.18 | 0.48 |  |  |
| Well-Being | -0.49 |  | 0.57 |  |
| Checking |  | 0.85 |  |  |
| Ordering |  | 0.75 |  |  |
| Cleaning |  | 0.60 |  |  |
| Claustrophobia |  | 0.67 |  |  |
| Euphoria |  |  | 0.89 |  |
| Covariates | | | |  |
| Cov resid (Appetite Loss - Appetite Gain) |  | -0.53 |  |  |
| Cov (Distress - Obsessions/Fear) |  | 0.61 |  |  |
| Cov (Distress - Positive Mood) |  | -0.18 |  |  |
| Cov (Obsessions/Fear - Positive Mood) |  | 0.34 |  |  |

***Note.*** CFA was conducted using the Maximum Likelihood method, with the Satorra–Bentler estimator. Values represent standardized *β* coefficients. Cov = covariates; resid = residuals; coefficients are significant at p < .001; *N* = 212

**Goodness of fit:**

TLI: 0.923

CFI: 0.936

RMSEA: 0.073

SRMR: 0.064

Chi-square: 269.09, p < .001

Table S5

Gender differences – IDAS-II subscales

|  | Females | | | Males | | |  |
| --- | --- | --- | --- | --- | --- | --- | --- |
|  | N | M(SD) | Range | N | M(SD) | Range | *p* |
| General Depression | 106 | 65.74 (17.17) | 22-94 | 106 | 53.02 (17.54) | 24-96 | <.001* |
| Dysphoria | 106 | 34.1 (10.22) | 10-50 | 106 | 27.8 (10.23) | 10-50 | <.001* |
| Lassitude | 106 | 21.57 (6) | 6-30 | 106 | 16.95 (5.76) | 6-30 | <.001* |
| Ill Temper | 106 | 16.29 (5.78) | 5-25 | 106 | 12.48 (5.49) | 5-25 | <.001* |
| Panic | 106 | 23.29 (9.76) | 8-40 | 106 | 17.2 (8.51) | 8-39 | <.001* |
| Traumatic Intrusions | 106 | 12.06 (4.7) | 4-20 | 106 | 9.8 (4.87) | 4-20 | <.001* |
| Insomnia | 106 | 18.35 (6.08) | 6-30 | 106 | 14.92 (5.55) | 6-28 | <.001* |
| Appetite Loss | 106 | 8.57 (3.9) | 3-15 | 106 | 6.86 (3.63) | 3-15 | .001* |
| Mania | 106 | 14.44 (4.63) | 5-24 | 106 | 12.83 (4.63) | 5-25 | .015 |
| Suicidality | 106 | 11.82 (6.51) | 6-30 | 106 | 9.67 (5.44) | 6-28 | .010 |
| Traumatic Avoidance | 106 | 12.08 (4.54) | 4-20 | 106 | 11.6 (4.14) | 4-20 | .470 |
| Appetite Gain | 106 | 7.83 (3.7) | 3-15 | 106 | 7.41 (3.17) | 3-15 | .561 |
| Cleaning | 106 | 15.3 (7.73) | 7-35 | 106 | 13.33 (6.15) | 7-34 | .119 |
| Ordering | 106 | 12.94 (4.7) | 5-24 | 106 | 11.92 (4.72) | 5-25 | .105 |
| Checking | 106 | 9.14 (3.43) | 3-15 | 106 | 8.27 (3.3) | 3-15 | .080 |
| Claustrophobia | 106 | 9.62 (5.4) | 5-22 | 106 | 7.39 (3.84) | 5-21 | .002* |
| Social Anxiety | 106 | 18.66 (6.92) | 6-30 | 106 | 14.52 (6.21) | 6-30 | <.001* |
| Euphoria | 106 | 10.5 (4.42) | 5-25 | 106 | 12.27 (4.09) | 5-22 | .001* |
| Well-Being | 106 | 21.09 (6.47) | 10-36 | 106 | 24.35 (6.64) | 8-39 | <.001* |

***Note.*** *N* = 212; between-group comparisons were conducted using the Mann–Whitney *U* test; starred comparisons were considered significant at Bonferroni corrected *α* < .003

Table S6.

Group comparisons by age – IDAS-II subscales

|  | Participants aged 19-44 | | | Participants aged 45-65 | | |  |
| --- | --- | --- | --- | --- | --- | --- | --- |
|  | N | M(SD) | Range | N | M(SD) | Range | p |
| General Depression | 58 | 68.21 (17.14) | 22-94 | 58 | 42.88 (14.27) | 23-81 | <.001* |
| Dysphoria | 58 | 35.47 (9.87) | 10-50 | 58 | 21.57 (8.82) | 10-39 | <.001* |
| Lassitude | 58 | 22.45 (5.73) | 6-30 | 58 | 12.84 (5.27) | 6-26 | <.001* |
| Ill Temper | 58 | 16.05 (6.02) | 5-25 | 58 | 9.98 (3.85) | 5-19 | <.001* |
| Panic | 58 | 24.14 (9.65) | 8-39 | 58 | 14.41 (7.19) | 8-35 | <.001* |
| Traumatic Intrusions | 58 | 12.66 (4.86) | 4-20 | 58 | 7.36 (3.67) | 4-18 | <.001* |
| Insomnia | 58 | 18.72 (6.03) | 6-30 | 58 | 14 (6.39) | 6-30 | <.001* |
| Appetite Loss | 58 | 8.79 (4.06) | 3-15 | 58 | 5.55 (2.91) | 3-15 | <.001* |
| Mania | 58 | 14.84 (4.05) | 6-24 | 58 | 10.72 (3.55) | 5-18 | <.001* |
| Suicidality | 58 | 13 (6.65) | 6-30 | 58 | 7.16 (1.55) | 6-12 | <.001* |
| Traumatic Avoidance | 58 | 12.36 (3.87) | 4-20 | 58 | 9.81 (4.19) | 4-20 | .001* |
| Appetite Gain | 58 | 7.76 (3.79) | 3-15 | 58 | 5.47 (2.47) | 3-15 | .001* |
| Cleaning | 58 | 15.84 (7.52) | 7-35 | 58 | 13.38 (6.02) | 7-28 | .069 |
| Ordering | 58 | 12.86 (4.35) | 5-24 | 58 | 10.84 (3.72) | 5-20 | .014 |
| Checking | 58 | 9.21 (3.19) | 3-15 | 58 | 5.86 (2.45) | 3-12 | <.001* |
| Claustrophobia | 58 | 9.26 (5.12) | 5-22 | 58 | 7.88 (3.52) | 5-17 | .352 |
| Social Anxiety | 58 | 18.86 (7.22) | 6-30 | 58 | 11.12 (4.09) | 6-22 | <.001* |
| Euphoria | 58 | 10.88 (4.27) | 5-21 | 58 | 11.53 (4.48) | 5-20 | .445 |
| Well-Being | 58 | 20.28 (6.59) | 10-39 | 58 | 25.52 (6.64) | 11-37 | <.001* |

***Note.*** The analysis above was conducted on *N* = 116 based on the following selection: all available participants aged 45-65 (58 participants), together with an equal subsample of randomly selected participants aged 19-44 (seed for random selection: 36548292). Between-group comparisons were conducted using the Mann–Whitney *U* test; starred comparisons were considered significant at Bonferroni corrected *α* < .003

Table S7.

Confirmatory Factor Analysis for participants 19-44 years old

|  | Distress | Obsessions/Fear | Positive Mood | |
| --- | --- | --- | --- | --- |
| Dysphoria | 0.94 |  |  |  |
| Lassitude | 0.79 |  |  |  |
| Ill Temper | 0.72 |  |  |  |
| Panic | 0.86 |  |  |  |
| Traumatic Intrusions | 0.80 |  |  |  |
| Insomnia | 0.63 |  |  |  |
| Appetite Loss | 0.51 |  |  |  |
| Suicidality | 0.65 |  |  |  |
| Appetite Gain | 0.32 |  |  |  |
| Mania | 0.70 |  | 0.44 |  |
| Social Anxiety | 0.68 | 0.19 |  |  |
| Traumatic Avoidance | 0.14 | 0.48 |  |  |
| Well-Being | -0.46 |  | 0.64 |  |
| Checking |  | 0.81 |  |  |
| Ordering |  | 0.72 |  |  |
| Cleaning |  | 0.58 |  |  |
| Claustrophobia |  | 0.60 |  |  |
| Euphoria |  |  | 0.84 |  |
| Covariates | | | |  |
| Cov resid (Appetite Loss - Appetite Gain) |  | -0.49 |  |  |
| Cov (Distress - Obsessions/Fear) |  | 0.61 |  |  |
| Cov (Distress - Positive Mood) |  | -0.19 |  |  |
| Cov (Obsessions/Fear - Positive Mood) |  | 0.40 |  |  |

***Note.*** *N* = 1,006 (58 participants were excluded from this analysis, aged 45-65)**.** CFA was conducted using the Maximum Likelihood method, with the Satorra–Bentler estimator. Values represent standardized *β* coefficients. Cov = covariates; resid = residuals; coefficients are significant at *p* < .001

**Goodness of fit:**

TLI: 0.917

CFI: 0.931

RMSEA: 0.072

SRMR: 0.056

Chi-square: 786.55, *p* < .001

Table S8.

Comparisons on IDAS-II scores between groups of participants showing high and low scores on BDI and BAI

|  | BDI | | | | | | | BAI | | | | | | |
| --- | --- | --- | --- | --- | --- | --- | --- | --- | --- | --- | --- | --- | --- | --- |
|  | Mild |  | Moderate |  | Severe |  |  | Mild |  | Moderate |  | Severe |  |  |
|  | N | M(SD) | N | M(SD) | N | M(SD) | *p* | N | M(SD) | N | M(SD) | N | M(SD) | *p* |
| General Depression | 420 | 44.83 (12.44) | 362 | 66.26 (9.68) | 282 | 78.94 (9.34) | <.001 | 527 | 49.4 (14.7) | 266 | 67.71 (11.12) | 271 | 77.62 (10.47) | <.001 |
| Dysphoria | 420 | 22.34 (7.7) | 362 | 35.74 (6.23) | 282 | 42.56 (4.93) | <.001 | 527 | 25.23 (9.28) | 266 | 36.29 (6.59) | 271 | 41.99 (5.62) | <.001 |
| Lassitude | 420 | 15.14 (5.25) | 362 | 21.89 (4.17) | 282 | 24.69 (3.83) | <.001 | 527 | 16.59 (5.73) | 266 | 22.03 (4.31) | 271 | 24.49 (4.08) | <.001 |
| Ill Temper | 420 | 10.69 (4.59) | 362 | 16.41 (5.1) | 282 | 19.34 (4.94) | <.001 | 527 | 11.68 (5.2) | 266 | 16.8 (4.94) | 271 | 19.41 (4.83) | <.001 |
| Panic | 420 | 14.21 (6.21) | 362 | 23.67 (7.07) | 282 | 29.57 (7.1) | <.001 | 527 | 14.56 (5.63) | 266 | 24.42 (5.43) | 271 | 32.13 (5.45) | <.001 |
| Traumatic Intrusions | 420 | 7.67 (3.52) | 362 | 11.83 (4.1) | 282 | 15.13 (3.64) | <.001 | 527 | 8.13 (3.77) | 266 | 12.37 (3.71) | 271 | 15.48 (3.49) | <.001 |
| Insomnia | 420 | 13.2 (5.33) | 362 | 17.51 (5.26) | 282 | 20.55 (5.4) | <.001 | 527 | 13.84 (5.4) | 266 | 17.71 (5.08) | 271 | 20.93 (5.47) | <.001 |
| Appetite Loss | 420 | 6.31 (3.05) | 362 | 8.5 (3.53) | 282 | 9.84 (3.83) | <.001 | 527 | 6.48 (3.11) | 266 | 8.74 (3.61) | 271 | 10.21 (3.61) | <.001 |
| Mania | 420 | 11.2 (3.93) | 362 | 14.67 (4.25) | 282 | 16.46 (4.42) | <.001 | 527 | 11.36 (3.89) | 266 | 14.79 (4) | 271 | 17.48 (3.99) | <.001 |
| Suicidality | 420 | 7.19 (2.32) | 362 | 10.62 (4.86) | 282 | 16.29 (6.56) | <.001 | 527 | 7.91 (3.39) | 266 | 11.89 (5.69) | 271 | 15.23 (6.73) | <.001 |
| Traumatic Avoidance | 420 | 10.2 (4.19) | 362 | 12.51 (3.83) | 282 | 12.9 (4.01) | <.001 | 527 | 10.32 (4.15) | 266 | 12.54 (3.65) | 271 | 13.55 (3.88) | <.001 |
| Appetite Gain | 420 | 6.74 (3.11) | 362 | 8.26 (3.45) | 282 | 8.71 (3.8) | <.001 | 527 | 7.02 (3.26) | 266 | 8.37 (3.47) | 271 | 8.68 (3.75) | <.001 |
| Cleaning | 420 | 12.98 (5.82) | 362 | 14.79 (6.67) | 282 | 16.24 (7.33) | <.001 | 527 | 12.82 (5.82) | 266 | 14.99 (6.67) | 271 | 17.13 (7.24) | <.001 |
| Ordering | 420 | 10.95 (4.01) | 362 | 13.14 (4.69) | 282 | 13.16 (4.96) | <.001 | 527 | 10.96 (4.15) | 266 | 12.9 (4.47) | 271 | 14.25 (4.87) | <.001 |
| Checking | 420 | 6.92 (2.98) | 362 | 9.36 (2.99) | 282 | 10.2 (3.59) | <.001 | 527 | 7.21 (3.05) | 266 | 9.28 (3.05) | 271 | 10.7 (3.33) | <.001 |
| Claustrophobia | 420 | 7.33 (3.73) | 362 | 9.76 (5.17) | 282 | 12.31 (6.15) | <.001 | 527 | 7.12 (3.37) | 266 | 10.18 (5.17) | 271 | 13.35 (6.13) | <.001 |
| Social Anxiety | 420 | 12.45 (4.98) | 362 | 19.2 (5.62) | 282 | 22.89 (5.38) | <.001 | 527 | 13.62 (5.53) | 266 | 19.47 (5.49) | 271 | 23.17 (5.47) | <.001 |
| Euphoria | 420 | 12.22 (4.33) | 362 | 10.91 (4.18) | 282 | 9.41 (4.14) | <.001 | 527 | 11.38 (4.23) | 266 | 10.67 (4.31) | 271 | 10.69 (4.66) | .033 |
| Well-Being | 420 | 26.68 (5.63) | 362 | 20.93 (5.36) | 282 | 17.16 (4.99) | <.001 | 527 | 24.75 (6.31) | 266 | 20.46 (5.99) | 271 | 18.95 (5.87) | <.001 |

***Note.*** *N* = 1.064; BDI categories: Mild (scores <15); Moderate (15-30); Severe (>30); BAI categories: Mild (scores <22); Moderate (22-35); Severe (>36); One-way analyses of variance were run separately on IDAS-II scales between BDI and BAI groups; all but Euphoria (BAI groups) were significant at the adjusted *α*. Follow-up pairwise comparisons with Bonferroni correction showed significant differences between each pair (all *p* < .016), with the following exceptions: BDI – Traumatic Avoidance (Moderate vs Severe*, p* = .682); BDI – Appetite Gain (Moderate vs Severe, *p* = .288); BDI – Ordering (Moderate vs Severe, *p* = 1); BAI – Euphoria (Moderate vs Severe, *p* = 1); BAI – Appetite Gain (Moderate vs Severe, *p* = .905)
